# Intratendinous ganglion in the extensor digitorum communis

**DOI:** 10.1080/23320885.2020.1833335

**Published:** 2020-10-13

**Authors:** Yoichi Sugiyama, Kiyohito Naito, Kenji Goto, Nana Nagura, Kazuo Kaneko

**Affiliations:** a Department of Orthopaedics, Juntendo University School of Medicine Tokyo, Japan; b Department of Orthopaedic Surgery, Juntendo Tokyo Koto Geriatric Medical Center, Tokyo, Japan

**Keywords:** Ganglion, intratendinous ganglion, extensor tendons, tenosynovitis

## Abstract

We encountered a patient with intratendinous ganglion which developed in the extensor digitorum communis tendon. Although its developmental mechanism is unclear, synovitis is considered the cause. For treatment, it may be necessary to prepare for tendon transfer and tendon grafting in consideration of the risk of tendon injury.

## Introduction

Ganglion is a benign tumor frequently encountered in routine medical practice. It has a cystic wall comprised of loose fibrous connective tissue and contains hyaluronic acid and mucopolysaccharides [1]. Ganglion is the most frequently developing benign tumor in the hands [2] and its frequent development site has been reported to be the scapholinate ligament of the dorsum of the hand accounting for about 70%, radiocarpal joint in the palm accounting for about 20%, and A1 pulley of the flexor tendon accounting for about 10% [3].

On the other hand, intratendinous development of ganglion is a very rare pathological state and fewer cases have been reported [4]. We encountered a patient with intratendinous ganglion which developed in the extensor tendon. We report the case including discussion about the developmental mechanism based on the surgical findings.

## Case report

**Figure 1.**
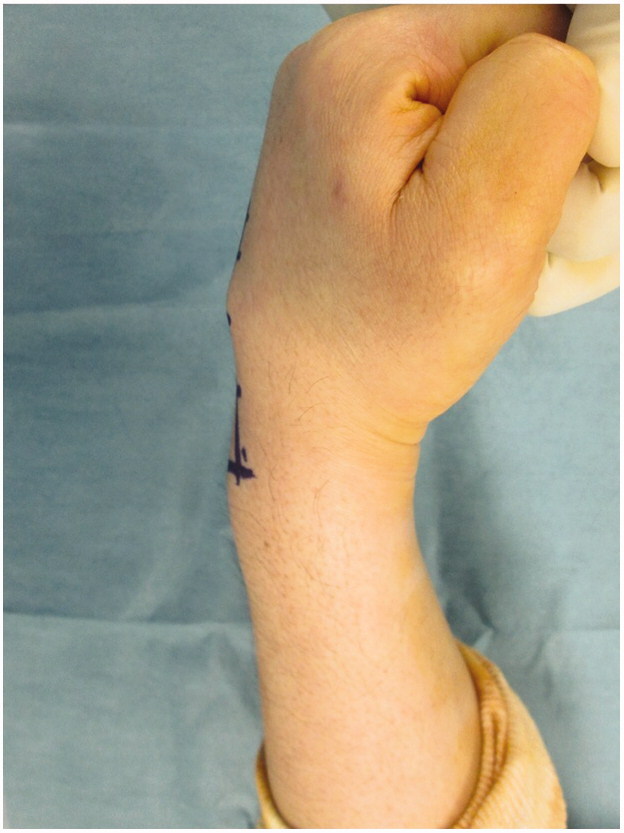
Mass in the dorsum of the hand. A mobile elastic hard mass with no tenderness was present in the proximal 3rd metacarpal bone of the dorsum of the left hand.

**Figure 2.**
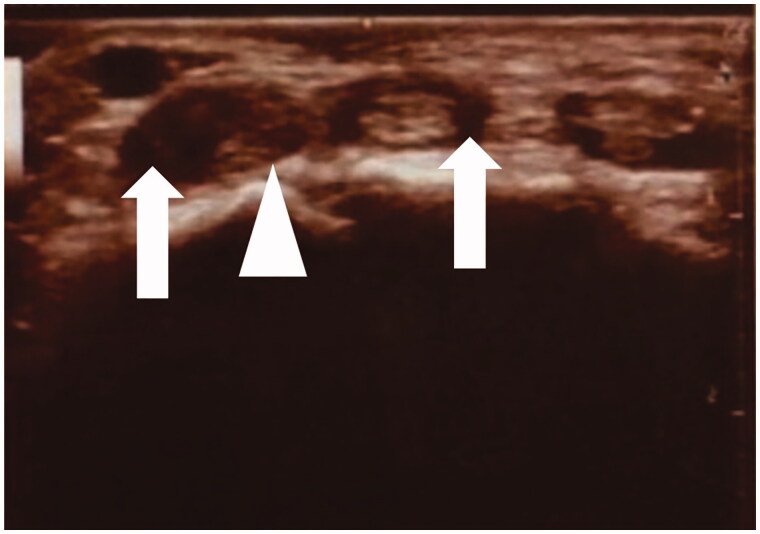
Preoperative diagnostic ultrasound imaging. A low echoic region (arrows) suggesting liquid was noted around the extensor digitorum communis (EDC) tendon and the 4th EDC tendon was not clearly visualized (arrow head).

**Figure 3.**
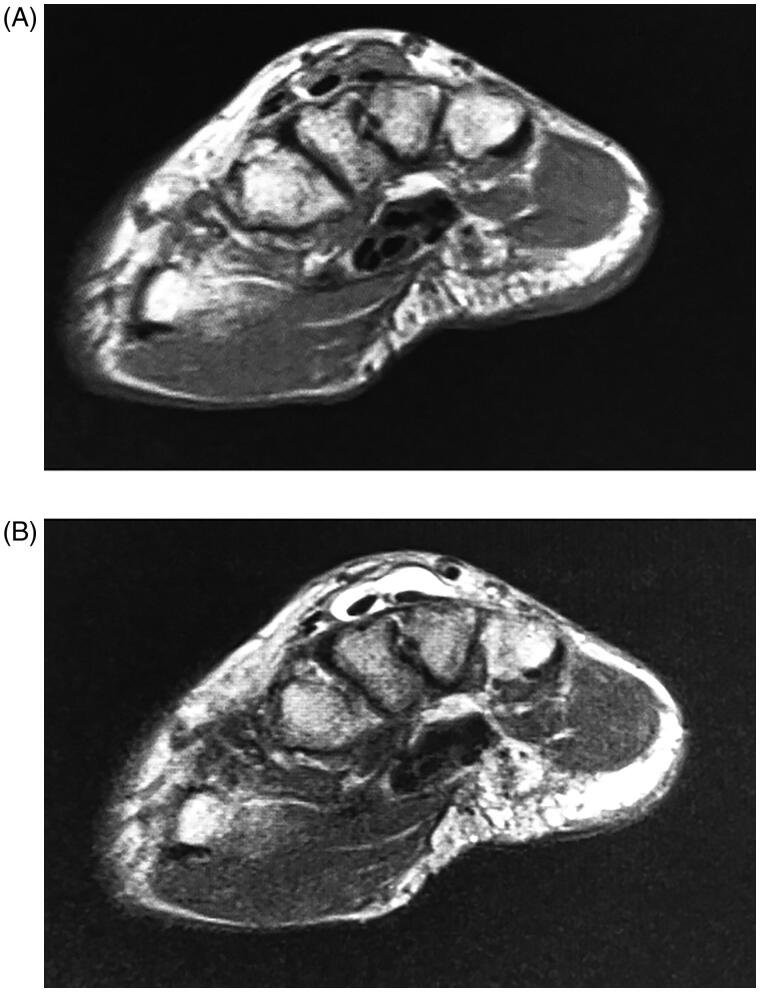
Preoperative axial MRI. At the metacarpal bone base level, lesions of approximately 8.3mm around the 3rd extensor digitorum communis (EDC) tendon and 11.3 mm around 4th EDC tendon were noted. Each showed a low-intensity lesion on T1-weighted imaging (A) and a high intensity on T2-weighted imaging (B).

The patient was an originally healthy 47-year-old male. He became aware of a mass present in the dorsum of the left hand several months earlier. The mass occasionally shrank, but it had not shrunk for one month before visiting our hospital. On the first visit, there was no pain of the wrist joint in motion or limitation of movement of the fingers. However, extension of the left middle and ring fingers caused tension of the skin of the dorsum of the hand and the patient was aware of difficulty in extension. The range of motion of the wrist joint was 70° of flexion and 85° of extension. The preoperative Q-DASH was 11.36/100, and VAS scores was 3/10. An elastic hard mass was palpated in the proximal metacarpal bone of the 3rd finger in the dorsum of the left hand and this mass had favorable mobility with the skin along with movement of the fingers (Figure 1). No osseous lesion was noted on plain radiography, a low echoic region suggesting the presence of liquid around the extensor digitorum communis (EDC) tendon was noted on diagnostic ultrasound imaging, and the 4th EDC tendon was not clearly visualized (Figure 2). On MRI, the lesions were approximately 8.3 mm in size around 3rd EDC and 11.3 mm in size around 4th EDC, with low intensity tumor lesions on T1-weighted images and high intensity tumor lesions on T2-weighted images (Figure 3(A, B)). Based on these findings, ganglion arising from the EDC tendon sheath was suspected and a definite diagnosis by excisional biopsy was planned.

**Figure 4.**
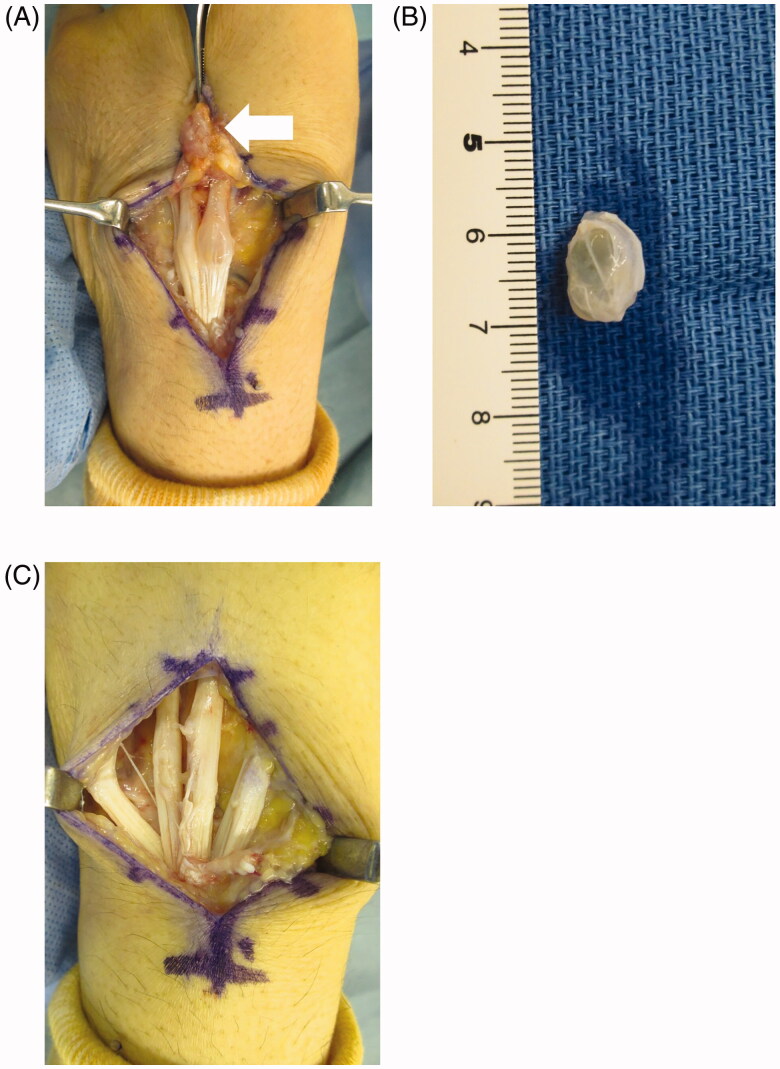
Surgical findings. (A) Synovial membrane outgrowth was observed around the tendon (arrow) and ganglion was present in the tendon of the 3rd and 4th EDC. (B) The tendon tissue was partially resected with the ganglion to remove the intratendinous ganglion. (C) The extensor tendon was partially injured after resection of the intratendinous ganglion, but tendon reconstruction was not necessary.

**Figure 5.**
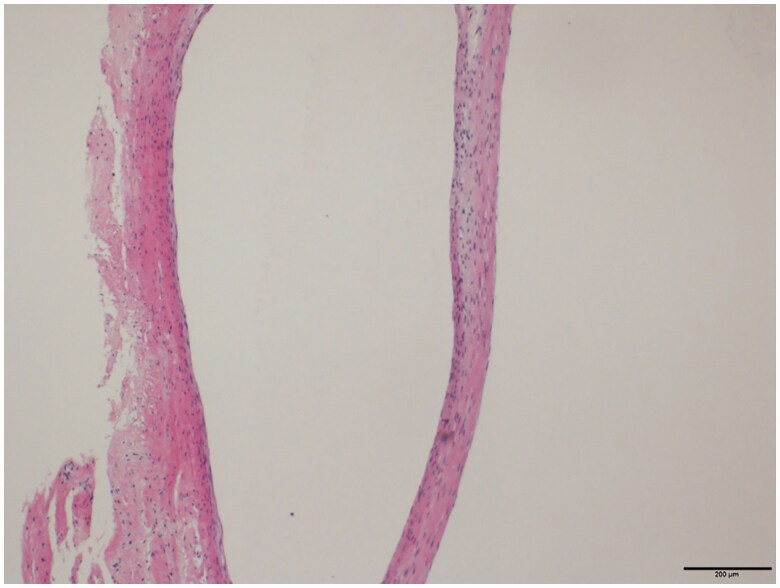
Pathological findings of the resected specimen. Cyst formation surrounded by fibrous connective tissue was noted, being diagnosed as ganglion.

In surgery, a longitudinal skin incision was made right above the tumor, and the extensor retinaculum was partially dissected from the distal end, reaching into the 4th compartment. Outgrowth of the synovial membrane was noted around the EDC tendon and the synovial membrane tissue was carefully dissected and resected from the EDC tendon. The ganglion was located in the tendons of the 3rd and 4th EDC (Figure 4(A)). Moreover, aberrant synovial membrane tissue was present in the EDC tendon. The excised ganglion was attached to a part of the EDC tendon (Figure 4(B)). The EDC tendon injuries were minimal (Figure 4(C)). Partial extensor tendon lacerations in zones II, IV, VI-VIII of the fingers have been reported to allow early active motion without the use of splints or sutures [5], so we did not suture the EDC tendon in this case. On histopathological examination, cyst formation surrounded by fiber tissue was noted, being diagnosed as intratendinous ganglion (Figure 5). As of one year after surgery, no recurrence of the ganglion or tear or adhesion of the EDC tendon has occurred. No contracture of the fingers has occurred, the range of motion of the wrist joint was 85° of flexion and 85° of extension, being graded as 2/10 VAS and a Q-DASH score of 6.82/100. The patient had returned to daily life without any problem.

## Discussion

Among benign tumors developing from the dorsum of the hand, ganglion developing from the scapholunate ligament is the most prevalent [6]. In this patient, ganglion was suspected based on the presence of a tumor with a clearly observed liquid component in the tendon on MRI, but it was not the most prevalent ganglion developing from the scapholunate ligament. Thus, excisional biopsy was performed to make a definite diagnosis.

The developmental mechanism of an intratendinous ganglion is unclear, but Seidman and Margles mentioned that it secondarily develops after synovitis [7], and Senda et al. mentioned that chronic stimulation, such as friction with the extensor retinaculum and metacarpal bosses, is the cause [4]. In our patient, no metacarpal boss was noted, the development site was the distal extensor retinaculum, and outgrowth of the surrounding synovial membrane was noted during surgery, suggesting that the cause was synovitis. In previous case reports of intratendinous development of ganglion, repeated trauma-induced mucous degeneration of the tendon and infiltration of tendonitis-induced inflammatory synovial membrane into the tendon parenchyma were considered the causes [7,8]. Outgrowth of the synovial membrane around the tendon and the presence of aberrant synovial membrane tissue in the tendon tissue were noted in our patient, suggesting that development of synovial membrane-derived ganglion in the tendon tissue was the developmental mechanism.

Treatment of ganglion includes puncture and resection [9], but an increase in the risk of tendon injury in the future was predicted in this synovitis-associated case because synovial membrane outgrowth was observed around the tendon on preoperative diagnostic ultrasound imaging [10], which may be difficult to treat with conservative treatment with puncture because of a high likelihood of frequent recurrence. Accordingly, puncture was excluded from the treatment options. Actually, the intratendinously developing ganglion entered the tendon fibers in this patient, for which resection of the ganglion including a part of the tendon was selected. Tendon reconstruction after ganglion resection was not necessary in this patient, but it may be necessary depending on the case. Preparation for tendon transfer and grafting may be necessary in consideration of the risk for tendon injury based on the size and location of the ganglion on preoperative imaging and preoperative evaluation of synovial membrane outgrowth around the tendon.

We encountered a patient with rare intratendinous ganglion which developed in the extensor tendon. Aberrant intratendinous synovial membrane tissue was considered a cause of intratendinous development of ganglion based on the surgical findings. Since tendon reconstruction after resection may be necessary when ganglion developing around and in the tendon is resected, preparation and informed consent for reconstructive surgery may be necessary.
